# Supplementary material for: Thermally active nanoparticle clusters enslaved by engineered domain wall traps
Source: Nat Commun. 2021 Oct 4;12:5813. doi: 10.1038/s41467-021-25931-7 (PMC8490384; doi:10.1038/s41467-021-25931-7)
Supplement: Supplementary file 3 — Description of Additional Supplementary Files [file 41467_2021_25931_MOESM3_ESM.pdf]

Description of Additional Supplementary Movie Files

File Name: Supplementary Movie 1

Description: This videoclip illustrates the real time dynamics of magnetic nanoparticles having a size of 360 nm and trapped by a magnetic bubble lattice. The magnetic bubbles are visible due to the polar Faraday effect, and they appear in the video as white disks immersed in a grey film. The FGF film is not subjected to any external field. The video corresponds to Figure 1(c) of the article.

File Name: Supplementary Movie 2

Description: This videoclip illustrates the escape dynamics of the magnetic nanoparticles when the FGF film is subjected to an external field with inverted direction (-z). The applied field has amplitude of 4mT and is applied at time t=2.3s. due to the applied field, the magnetic energy landscape inverts, and the nanoparticle escape from the bubble accumulating above the interstitial regions. After 4.6s the magnetic field is inverted again, and the nanoparticles come back to the same or a different magnetic domain. The video corresponds to Figure 6(a) of the article.
